# Immune responses elicited by ssRNA(−) oncolytic viruses in the host and in the tumor microenvironment

**DOI:** 10.20517/2394-4722.2022.92

**Published:** 2023-04-04

**Authors:** Yonina Bykov, Gloria Dawodu, Aryana Javaheri, Adolfo Garcia-Sastre, Sara Cuadrado-Castano

**Affiliations:** 1 Department of Microbiology, Icahn School of Medicine at Mount Sinai, New York, NY 10029, USA.; 2 Department of Medicine, Icahn School of Medicine at Mount Sinai, New York, NY 10029, USA.; 3 Global Health and Emerging Pathogens Institute, Icahn School of Medicine at Mount Sinai, New York, NY 10029, USA.; 4 The Tisch Cancer Institute, Icahn School of Medicine at Mount Sinai, New York, NY 10029, USA.; 5 Department of Pathology, Molecular and Cell-Based Medicine, Icahn School of Medicine at Mount Sinai, New York, NY 10029, USA.

**Keywords:** Oncolytic virus, NDV, IAV, virotherapy, paramyxovirus, orthomyxovirus, ssRNA(−), immunotherapy, cancer vaccine, ICD, *in situ* vaccination, tumor microenvironment

## Abstract

Oncolytic viruses (OVs) are at the forefront of biologicals for cancer treatment. They represent a diverse landscape of naturally occurring viral strains and genetically modified viruses that, either as single agents or as part of combination therapies, are being evaluated in preclinical and clinical settings. As the field gains momentum, the research on OVs has been shifting efforts to expand our understanding of the complex interplay between the virus, the tumor and the immune system, with the aim of rationally designing more efficient therapeutic interventions. Nowadays, the potential of an OV platform is no longer defined exclusively by the targeted replication and cancer cell killing capacities of the virus, but by its contribution as an immunostimulator, triggering the transformation of the immunosuppressive tumor microenvironment (TME) into a place where innate and adaptive immunity players can efficiently engage and lead the development of tumor-specific long-term memory responses. Here we review the immune mechanisms and host responses induced by ssRNA(−) (negative-sense single-stranded RNA) viruses as OV platforms. We focus on two ssRNA(−) OV candidates: Newcastle disease virus (NDV), an avian paramyxovirus with one of the longest histories of utilization as an OV, and influenza A (IAV) virus, a well-characterized human pathogen with extraordinary immunostimulatory capacities that is steadily advancing as an OV candidate through the development of recombinant IAV attenuated platforms.

## INTRODUCTION

Documented cases of viral illnesses sporadically clearing cancer in patients have sparked research into how viruses could be utilized as cancer therapeutics since the start of the twentieth century, with the greatest boom in developing and optimizing viral vectors occurring within the past two decades^[1–4]^. The establishment and optimization of reverse genetics systems for both DNA and RNA viruses has been the major driver in advance of virotherapy, thus allowing for the design of safer and targeted viruses and further understanding of the therapeutic capabilities of different OVs platforms. As of today, only four OVs have been granted approval for clinical application: an enterovirus ECHO-7-based virotherapy [Enteric Cytopathogenic Human Orphan-7; ssRNA(+) virus] was the first OV platform registered and approved for the treatment of melanoma in Latvia, later on, followed by Georgia, Uzbekistan and Armenia authorizations^[5]^. Soon after, in 2005, the Chinese Food and Drug Administration Agency (CFDA) gave the green light to Oncorine^®^ (H101, dsDNA virus), a modified human adenovirus that, in combination with chemotherapy, became the first immunotherapy for patients with refractory head and neck carcinoma^[6]^. In 2015, T-VEC (Talimogene laherparepvec; IMLIGYC^®^), a recombinant human Herpes virus (HSV-1, dsDNA virus) expressing GM-CSF (granulocyte-macrophage colony-stimulating factor) was approved by the US Food and Drug Administration (FDA) for the local treatment of recurrent, unresectable melanoma in adult patients that could not undergo further surgery. T-VEC is now also approved in Europe, Australia and Israel^[7]^. DELYTACT^®^ (Teserpaturev/G47Δ), also an HSV-1 viral platform, is the most recently approved OV immunotherapy (Japan, 2021) and the first indicated for treating gliomas or any primary brain cancer^[8]^.

**Figure 1.**
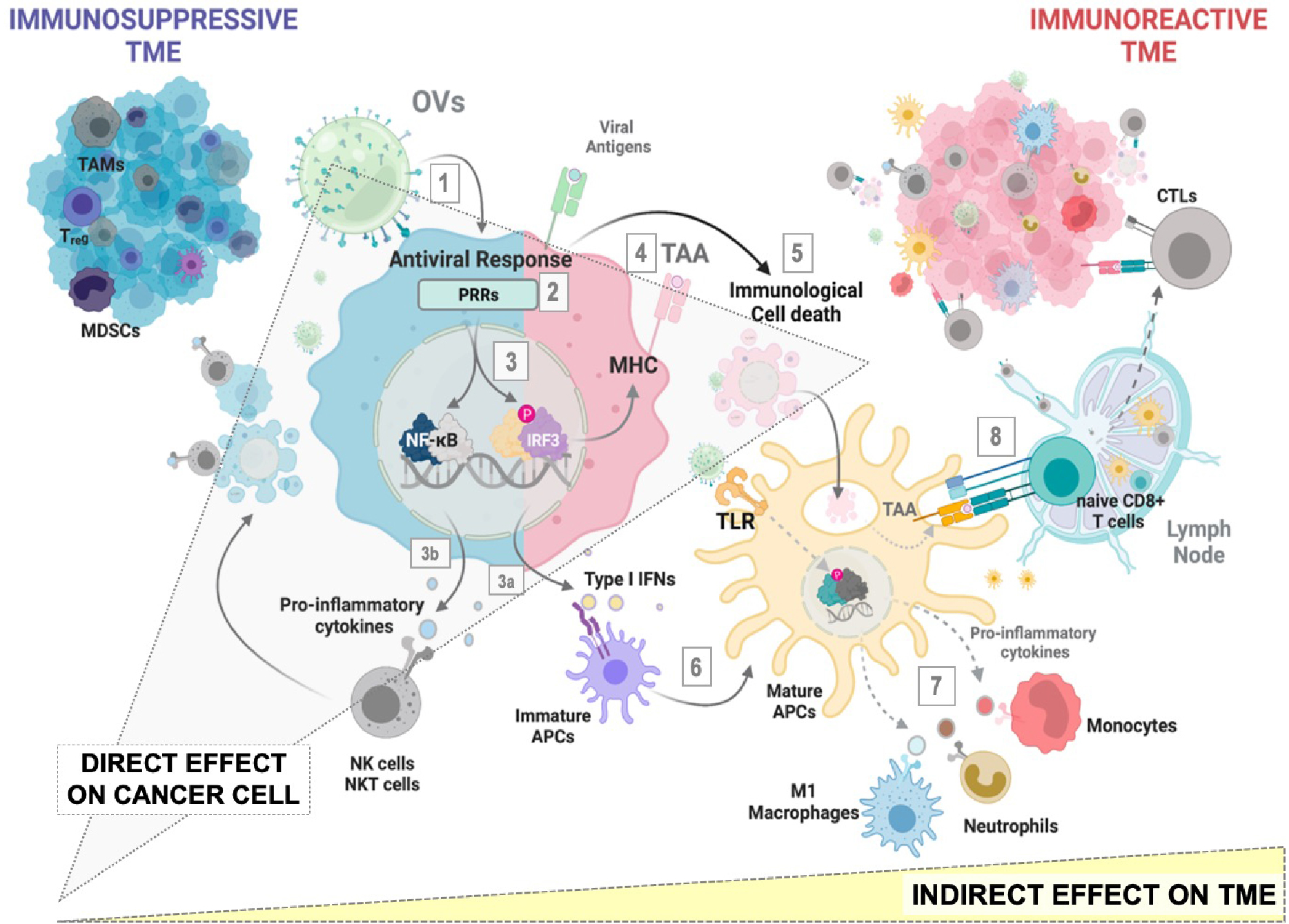
Direct and Indirect effects of OVs at the tumor site. Direct effect^[1–5]^: infection of cancer cells^[1]^ leads to the recognition of viral genomes by PRRs^[2]^ and subsequent activation of signaling pathways involved in antiviral defense^[3,4]^; Type-I interferons and proinflammatory cytokines released to the TME together with DAMPS activation of cell death mechanisms^[5]^ serve as attractants and activators of the innate compartment. Indirect effect^[6–8]^: licensed APCs cross-present TAA to naïve T-cells^[8]^, allowing for the generation of tumor-specific cytotoxic CD8^+^ T cells. MDSCs: myeloid-derived suppressor cells; TAMs: Tumor-associated Macrophages; T-regs: regulatory T cells; APC: antigen-presenting cell; OVs: Oncolytic viruses; PRRs: Pattern Recognition Receptors; MHC: Mayor Histocompatibility complex; TAA: tumor-associated antigen; NK: natural killer; NKT: Natural Killer T cells; IRF3: interferon regulatory factor 3; TLR: toll-like receptor; CTL: cytotoxic T-lymphocytes; TME: tumor microenvironment.

The actual landscape of OVs candidates being explored in preclinical and clinical settings reflects the interest and high expectation from the oncological community in the applicability of virotherapy for cancer^[9]^. Regardless of viral classification, host range or replication capacity, a therapeutic OV candidate will lead to the induction of antitumor response through a three-pronged approach: (1) Infection of cancer cells, leading to the activation of antiviral mechanisms and regulated cell death (RCD) pathways; (2) Recruitment of innate immune mediators and antigen-presenting cells (APCs) to the tumor microenvironment, where damage-associated (DAMPs) and pathogen-associated molecular patterns (PAMPs), as well as tumor (TA) and tumor-associated antigens (TAA) contribute to APC licensing; and (3) Cross presentation of TA and TAA to CD8+ cytotoxic T-cells, allowing for a systemic tumor-specific response^[10]^. Therefore, the impact of OVs extends beyond the infected cancer cells, leading the way to remodeling the composition of the TME and breaking the immunotolerance that allows for cancer progression: tumors escape clearance by the immune system by promoting an immunosuppressive TME, characterized by a state of chronic inflammation and suppressive immune populations, thus including myeloid-derived suppressor cells (MDSCs), tumor-associated macrophages (TAMs), and regulatory T cells (Tregs). OV-infected cells upregulate type I IFNs and secrete proinflammatory cytokines resulting in the recruitment and activation of innate immune subsets such as Natural Killer (NK) cells, M1-like macrophages and dendritic cells. Additionally, activated T cells recruited to the tumor are more likely to be functional in the increasingly immune-activated tumor milieu. These changes can be summarized as a conversion into an immunoreactive TME that promotes the clearance of treated tumors [Figure 1]^[11]^.

**Figure 2.**
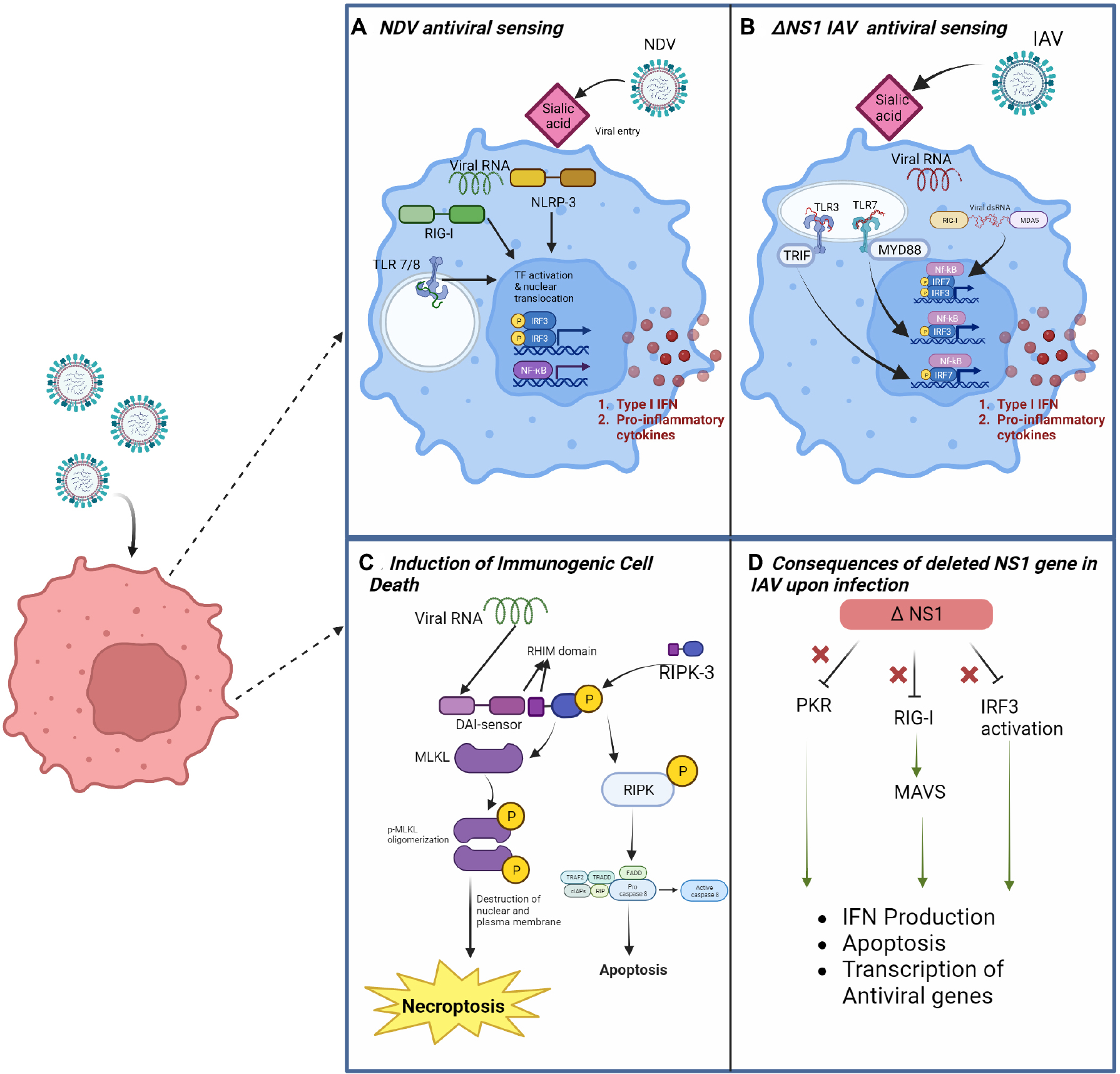
Intracellular pathways involved in the oncolytic activity of NDV and Influenza A Viruses. (A) Schematic of viral RNA sensing by NLRP-3 nod-like receptors and RIG-I-like receptors in the cytosol as well as TLR7/8 in endosomes containing viral RNA. Detection leads to activation of NF-kβ and IRF3 and translocation of these transcription factors to the nucleus, followed by production of type 1 interferons and proinflammatory cytokines. (B) In addition to RIG-I, IAV viral RNA could be sensed by the RLR Melanoma Differentiation-Associated gene 5 (MDA-5), and by TLR3/7. (C) Viral RNA sensing mediated by a DAI (DNA-dependent activator of IFN-regulatory factors) sensor, leading to activation of RIPK-3 and downstream MLKL (mixed lineage kinase domain-like pseudokinase) and resulting in cell death pathways such as necroptosis and apoptosis. (D) Illustration of viral response inhibition mediated by the NS1 gene of IAV. In the absence of NS1, activation of PKR, RIG-I, and IRF3 leads to upregulation of pathways necessary for antiviral response.

On the whole, the upshot of cancer virotherapy relies on the direct effect that the virus exerts over the cancer cell and the subsequent indirect effect that the cellular response to the infection has on potentiating innate and adaptive immune responses in an otherwise immunosuppressive TME^[12]^. Knowing how a particular cancer type responds to a particular OV is, therefore, of high relevance to rationally designing tumor-specific targeted virotherapies. In this regard, successful OVs are those more suitable to trigger cellular stress that resolves in the form of immunogenic cell death (ICD) [Figure 2]: the activation of RCD programs and release of associated endogenous DAMPs (nucleic acids, ATP, ecto-CRT, HMGB, and others), in conjunction with antiviral signaling products - viral PAMPS, type-I interferons, cytokines-resolves in antigen-specific effector and memory T cell functions^[13]^. In other words, ICD responses to OVs therapies provide the antigenicity and adjuvanticity needed to drive tumor elimination and long-term protection.

ssRNA(−) viruses have been gaining popularity as oncolytic vectors over the last few decades as we have deepened our understanding of their biology, virus-host interactions and potential strengths various viral subclasses may have as therapeutics. Overall, ssRNA(−) viruses have been shown to be robust immunostimulators of innate immunity, starting with the activation of type I IFN signaling cascades in the infected cancer cells that further translates into activation and remodeling of the myeloid population at the TME^[14]^. Though immune responses to these viruses share some similarities, each has its own strengths that may be of value when evaluating which oncolytic virus to utilize for a particular type of cancer [Table 1].

In this review, we highlight two negative-sense RNA viruses that hold great potential for clinical use: Newcastle Disease Virus (NDV), a well-studied avian paramyxovirus, and Influenza A, which is less explored as an oncolytic, but promising due to its potent induction of immunogenic cell death that can be attenuated accordingly, as evidenced by the large-scale production and use of seasonal IAV vaccines. Though these viruses are from different families, the processes by which they are sensed within the innate immune system have significant similarities. Both viruses are recognized by RIG-I and MDA-5 RIG-I-like receptors (RLRs) within the cytoplasm, NLRP-3 nod-like receptors (NLRs) within the cytosol, and Toll-like receptors (TLR-s) 3 (for dsRNA) and 7 (for ssRNA) within the endosome. RIG-I mediated recognition triggers stimulation of MAVS, which leads to downstream production of type 1 IFN and transcription of interferon-stimulated genes (ISGs)^[63]^. Binding of RLRs and TLR-s to viral RNA triggers a signaling cascade that results in the activation and nuclear translocation of NF-kβ and IRFs, and the subsequent induction of proinflammatory signaling and antiviral programs^[64]^.

Although there are similarities in certain immune responses triggered by administration of NDV and influenza, there are differences in their interactions with both infected cells and the host immune response that influence their efficacy in distinct tumor types. As we describe next, each virus may be uniquely capable of mounting an exceptionally strong and safe response against certain cancers.

## NEWCASTLE DISEASE VIRUS AS AN ONCOLYTIC THERAPY

NDV has shown great promise as an oncolytic agent in numerous preclinical and clinical studies^[65–67]^. This non-segmented, enveloped virus of the *Paramyxoviridae* family has been isolated from various species of fowl worldwide^[68]^. Strains are broadly classified according to their virulence in chickens, as this is the species in which NDV is most frequently found. While there are multiple determinants of virulence, the viral F protein cleavage site is usually used as a primary genetic indicator. Lentogenic NDV strains, or strains with low virulence, typically have a monobasic cleavage site in the F protein and would require the addition of exogenous trypsin to infect neighboring cells. This reduces the ability of the virus to replicate in its host^[69]^. Velogenic, or highly virulent strains, have a polybasic cleavage site in the F protein that is recognized by ubiquitous furin-like host proteases, thus providing a significant replication advantage to the virus^[69]^. Mesogenic strains are of an intermediate virulence, but also tend to have a polybasic cleavage site^[70]^. NDV does not cause significant illness in humans, but genetic determinants of virulence in its natural host can affect mammalian tumor and host immune responses and are thus important to consider. Furthermore, lentogenic NDV viral platforms are more likely to be translated to the clinic due to concern over the potential (though the highly unlikely) spread of velogenic strains to domestic fowl.

NDV’s direct effect involves activation of the type I interferon response, cytokine release and induction of various RCD pathways (including apoptosis, necroptosis, necrosis, autophagy, and ferroptosis) depending on the tumor model and NDV strain^[71]^. The virus binds to sialic acid to infect cells, which means that it has a very broad tumor tropism (as opposed to several other candidate oncolytic viruses that are not discussed here). The potent induction of type I interferon is another major advantage of this platform. The indirect effects of NDV as OV are of great interest because they can (1) continue to promote tumor regression even after the virus is cleared by infected tumor cells or the host immune system; (2) are responsible for the abscopal effect (clearance of non-infected tumors); and (3) can provide long-term protection against recurrence^[23]^.

### NDV’s direct effects

The antitumor effects of NDV begin with the infection of cancer cells. Viral RNA from NDV is usually recognized by RIG-I in the cytosol of infected cells^[72–74]^. Viral 5′ triphosphate RNA binds strongly to RIG-I to promote downstream effects^[45]^. RLRs signal through the IPS/MAVS adaptor protein to induce activation and translocation of IRF-3, IRF7 and NF-kβ into the nucleus^[75,76]^. This results in transcription of antiviral genes and proinflammatory cytokines that signal in an autocrine and paracrine manner. NOD-like receptors (NLRs) can also play a role in recognition of viral RNA by infected cells. NDV has been shown to activate NRLP-3 in the THP1 human monocyte cell line^[15]^ and in chicken cells^[77]^, which results in the assembly of the inflammasome, caspase 1 cleavage and IL-1β secretion. Though it is unclear whether activation of NLRP3 can be generalized to both mammalian cancers and normal tissue, it is evident that NDV is a potent inducer of the type I IFN response in infected cells. Activation of the type I interferon response has multiple effects intended to counteract viral infection, including direct interference with viral replication, induction of inflammation in surrounding tissue, and induction of cell death^[78]^.

ICD: NDV can activate various RCDs depending on the infecting strain and the characteristics of the tumor. Apoptosis is one of the most frequently described pathways triggered by NDV infection^[16,79–81]^ and is relatively immunologically silent. Tumors with defects in pro-apoptotic signaling may undergo alternative modes of cell death in response to infection, including necrosis, necroptosis and pyroptosis. NDV expressing MIP-3a - a chemotaxic ligand for lymphocytes - has been shown to promote increased immunogenic cell death in B16 melanoma and 4T1 breast cancer cell lines, measured by increased levels of calreticulin at the cell surface and visualization of cell lysis by microscopy^[82]^. These features are indicative of necrosis, which is highly immunogenic and is accompanied by the release of large amounts of DAMPs and PAMPs. Several velogenic strains of NDV have been shown to cause necroptosis in human melanoma cell lines A375 and C8161^[19]^ and the cervical cancer cell line HeLa^[83]^. The mechanism involves localization of necrotic cell death machinery to stress granules in the cytoplasm instead of the membrane^[83]^ as is commonly observed during necrosis, though it remains to be seen whether this can be generalized to multiple cell types or an *in vivo* setting. Necroptosis and autophagy were observed both *in vitro* and *in vivo* in the GL261 murine glioblastoma model treated with the Hitchner B1 NDV lentogenic strain, translating to a potent *in vivo* immune response^[84]^.

#### Infection of immune cells

A vital component of the antitumor immune response is the activation of the innate compartment. This can occur through immunostimulatory signals from NDV-infected tumors, and also through infection of innate immune cells with NDV. NDV can infect a wide variety of cells due to its use of ubiquitous sialic acid residues for attachment to the host membrane. While it is unable to undergo multiple cycles of replication in healthy mammalian cells due to its susceptibility to the mammalian interferon response, it has been shown to have immunostimulatory effects on infected innate cells that in turn promote an enhanced antitumor effect. For instance, an attenuated strain of NDV has been shown to infect dendritic cells, monocytes and macrophages^[22]^. Burke *et al.* have shown that infection of human peripheral blood mononuclear cells (PBMCs) promotes secretion of the immunostimulatory cytokines IFN-α, IFN-γ, IL-6 and IL-8, a large portion of which comes from monocytes^[22]^. CD14+ monocyte-derived macrophages have an even more robust response to NDV infection, secreting high amounts of IFN, IL-6, IL-10 and TNF-α and producing toxic nitric oxide species even at low multiplicity of infection (MOI)^[22]^. In addition to secretion of proinflammatory cytokines, infected macrophages acquire a highly activated phenotype as measured by upregulation of HLA-DR, PD-L1 and the costimulatory receptor CD86. Interestingly, infected macrophages have been shown to release viral particles *in vitro* that can infect and kill nearby cancer cells^[22]^.

Dendritic cells, when infected with NDV, signal through RLRs and TLR-s, inducing activation and skewing towards a DC1 program. This is accompanied by upregulation of CD80, CD86, and CD40, as well as increased production of IFN-γ and TNF-α^[85–87]^. Increased differentiation of immature conventional DCs (cDC) into DC1 has been associated with improved response to immunotherapy and prolonged survival^[88]^. This is likely, at least in part, due to the potent ability of DC1 to activate naive CD4+ and CD8+ T cells. Furthermore, increased production of IFN-α in infected DCs can stimulate autocrine signaling that modulates the activity of the proteasome. Reduced proteolytic capacity of DC1 promotes cross-presentation to CD8+ T cells, which has been shown to correlate with better tumor control and prolonged survival^[88]^. IFN-α also promotes an anti-apoptotic program in pDC, leading to increased persistence of antigen-presenting cells in the tumor. Despite the above-described advantages of DC infection with NDV, there may also be inhibitory effects to consider. While infection with NDV promotes maturation of DCs^[22,85]^, it can also promote a more immunosuppressive phenotype measured by increased secretion of IL-10. Nan *et al.* reported increased TH_2_-like programming and reduced proliferation of T cells co-cultured with NDV-infected DCs^[85]^. Impaired proliferation was seen in both CD8+^[85]^ and CD4+^[85,89]^ T cells. Direct infection of DCs has not been studied in tumor-bearing mice, but if the above findings are also true in the context of cancer, it will be important to uncouple the immunostimulatory and immunosuppressive effects of NDV on DCs to achieve an optimal antitumor response.

### NDV’s indirect effects I: activation of the innate response by infected tumors

Infected tumors provide heightened immunostimulatory signals to innate cells. Infected cells secrete proinflammatory cytokines such as IFN-γ and IFN-α, and immunogenic cell death releases an assortment of PAMPS (comprised of viral proteins and RNA) and DAMPS (surface calreticulin, cellular DNA, ATP and more) to the surface or the extracellular milieu. Response to NDV treatment *in vivo* hinges on intact IFN-γ and IFN- αR, as blockade of either results in loss of therapeutic effect^[24]^. Signaling through both IFN-γ and IFN- αR promotes the recruitment and maturation of a wide array of immune cells.

DCs recruited to the tumor site efficiently take up PAMPs and DAMPs from lysed cancer cells through both macropinocytosis and clathrin-mediated endocytosis^[76]^. While antigens that are engulfed by macropinocytosis are presented on MHCII to CD4+ T cells via the classical pathway, those that enter via endocytosis are processed in endosomes to be cross-presented to CD8+ T cells on MHCI. In general, NDV treatment of tumors enhances dendritic cell maturation and activation. NDV oncolysate promotes the maturation of DCs, increasing the expression of the costimulatory ligands CD80, CD86 and CD83^[22,86,90]^. This allows for enhanced activation of T cells; T cells that are co-cultured with DCs loaded with viral oncolysate secrete high amounts of IFN-γ and IL-2. Different DC subsets have distinct responses to NDV. Plasmacytoid DCs express high levels of TLR-7, thereby secreting large amounts of interferon upon recognition of NDV’s RNA genome. TLR-bound ligands are conserved in endosomes and can stimulate prolonged IFN responses^[91]^. cDC1, dependent on IRF-1 for production of type I IFN, are thought to be more important for antitumor responses - primarily due to their unique adaptation for cross-presentation. Supernatants from tumors infected with NDV are also potent inducers of chemotaxis in DCs^[82]^.

The myeloid lineage also gives rise to CD11b+ MDSC. In healthy individuals, or in the context of acute infection, they can further differentiate into mature DC, granulocytes and macrophages. In the context of chronic inflammation, however, MDSCs are arrested in an immature, suppressive state. This population is associated with resistance to immunotherapy^[92]^ and is recruited by tumors. A variety of cancers can promote recruitment of MDSCs through secretion of the chemokines CCL2 and CCL5^[93]^, and promote their persistence via an array of immunosuppressive cytokines including TGF-β, IL-10 and soluble TNF-α^[94]^. MDSCs exert various suppressive functions, including inhibition of effector T cells, induction of Tregs, impairment of DC migration and maturation, and skewing of macrophages toward a pro-tumorigenic M2-like program^[93,94]^. NDV treatment has been shown to reduce the number of MDSCs in murine B16 melanoma^[82]^. Interestingly, the opposite effect was observed in the H22 murine model of HCC, possibly due to significant upregulation of IDO (Indoleamine 2,3-dioxygenase) and IL-6 by infected hepatocellular carcinoma cells^[95]^. This emerging evidence of differential effects of NDV in different tumor types indicates a need to develop a method to predict response to oncolytic virotherapy based on tumor intrinsic features and the tumor microenvironment to achieve a maximum probability of clinical success.

It is currently known that macrophages play a critical role in response to immunotherapy. While M2-like macrophages (CD206^+^ or Arginase I^+^) promote a state of chronic inflammation that is pro-tumorigenic and immunosuppressive, M1-like macrophages (MHC^hi^, NO^+^) have been shown to induce tumor regression and promote a more effective antitumor adaptive immune response. NDV treatment reduces the numbers of M2-like macrophages within murine B16 melanoma and 4T1 cervical cancer tumors^[82]^, though it is not entirely clear whether this is through a skewing of M2-like phenotypes to M1-like phenotypes, exclusion of M2-like macrophages from the tumor milieu, or expediting apoptosis of these macrophages. There is evidence suggesting the first option, as Wang *et al.* noted that macrophages infected with NDV produce NO downstream activation of NF-kβ signaling^[82]^. This is characteristic of an M1 program and promotes apoptosis of tumor cells.

Another major innate mediator of tumor clearance is natural killer (NK) cells. NK cells play a major role in both viral infection and early clearance of tumors. The balance of activating versus inhibitory signals encountered by NK cells determines whether they will be suppressed or will be activated to kill the target cell. NDV infection of tumor cells provides activating signals to NK cells via the interaction of the HN protein on the surface of infected cells with the NK receptors NKp46 and NKp44^[20,21]^. This interaction results in upregulation of TRAIL in NKs^[21]^, through which they may promote apoptosis of tumor cells. Enhanced cytotoxicity of NKs has been described *in vitro*^[96]^. Mouse studies demonstrate increased infiltration of NDV-treated B16 tumors with NK cells^[97]^, but *in vivo* phenotypic characterization of this cell type has not been done. As many tumors adapt to escape killing by NK cells (via secretion of inhibitory cytokines, shedding of activating ligands and presentation of inhibitory ligands^[98]^), activating signals derived from NDV can be a key factor in the reinvigoration of suppressed NKs. Indeed, NK cells have been shown to be important for *in vivo* efficacy of NDV treatment^[97]^.

### NDV’s indirect effects II: activation of the adaptive response by infected tumors

Treatment of tumors with NDV is also an effective strategy for activating the adaptive immune response. Although this is not consistent among all studies and all NDV strains, some NDV strains - especially in combination with therapeutic transgenes or immune checkpoint blockade impart long-term protection against re-challenge with the same tumor^[24,79,99]^. This is indicative of a robust T cell response coupled with effective and long-lasting memory. There are multiple ways in which NDV treatment can achieve this. Infected cells upregulate MHCI on the cell surface^[76,100]^, which means more antigens will be presented to cytotoxic CD8+T cells. The above-described inflammatory antiviral response induces the transcription factor IRF-1, which binds to the interferon response sequence of the MHCI gene promoter, thus inducing higher levels of transcription. NF-kβ, also induced by NDV infection, binds to the enhancer for MHCI^[76]^. Infected cells may therefore present more neoantigens that can stimulate a strong T cell response, or may present viral antigens^[101]^.

CD8+ T cells are vital for the full efficacy of NDV treatment, as antibody depletion *in vivo* leads to a significant reduction of therapeutic effect. While the depletion of CD4+ T cells slightly reduces the survival of treated mice, it appears that they are not essential for the efficacy of NDV^[97]^. Nonetheless, treatment of B16 melanoma tumors with NDV *in vivo* leads to an increase in infiltrating CD4+ and CD8+ T cells that are activated and highly proliferative^[23]^. This is true in both injected and distal tumors, and T cell infiltration is antigen-specific T-cell activation in the microenvironment of NDV-treated tumors is a complex event that can be difficult to deconvolute *in vivo*. *In vitro*, CD4+ T cells derived from the sTS3 T cell clone had increased proliferative capabilities when co-cultured with its autologous SMS melanoma tumor cell line infected with NDV-ulster, as compared to co-culture with uninfected SMS cells^[102]^. CD8+ T cells are also increasingly activated by NDV in culture-notably, the HN protein has been shown to promote CD8+ responses^[103]^. In a more recent study, Krabbe *et al.* similarly demonstrated that the co-culture of antigen-specific T cells with infected tumors promotes the activation of T cells (measured by increased PD-1, CD69 and CD25)^[100]^.

Tumor-infiltrating T cells consist of multiple populations with varying functionality, including FoxP3+ regulatory T cells (Tregs). Many studies have linked Tregs to resistance to immunotherapy^[104]^, and oftentimes tumors promote the differentiation of naive T cells into Tregs as one of many mechanisms of escape. Intratumoral treatment with NDV, as well as prior immunization, leads to a reduction of Tregs in the treated tumor^[82,97]^.

Another frequent immune evasion strategy and resistance mechanism by tumors is the upregulation of immunosuppressive molecules on the cell surface. Zamarin *et al*. identified that viral treatment of B16 melanoma tumors induces upregulation of PD-L1 on the tumor surface^[99]^. The suppressive effects of PD-L1 were blocked with aPD-1, establishing a synergistic effect with NDV. CTLA-4 blockade has also been shown to have a synergistic effect with NDV treatment, leading to the recruitment of greater numbers of activated and proliferating T cells and reduced numbers of Tregs as compared to either of the monotherapies^[24]^.

### Safety and clinical translatability of NDV platform

NDV has long been investigated in both preclinical and clinical studies, demonstrating an impressive safety profile. Lentogenic strains have been used as vaccine vectors for birds and for humans, with the most significant side effects in recent trials including mild injection site pain, fatigue, malaise, headache and occasionally fever^[105,106]^. Likewise, various clinical trials performed on cancer patients have shown that NDV induces very mild side effects, the most described being fatigue, headache and fever^[76,107]^. Adverse effects tend to resolve quickly and are much less severe than those observed with other modes of immunotherapy, marking NDV as a feasible and safe therapeutic for use in the clinic.

When considering the clinical translatability of NDV, the development of antiviral immunity is a potential concern that could limit the therapeutic effect of the virus. Preclinical work has shown, however, that the development of pre-existing immunity to NDV in mice does not hamper its efficacy and, notably, promotes a more robust antitumor immune response^[97]^. NDV has been tested in cancer patients as early as the 1964 report from Wheelock and Dingle, where a leukemia patient showed a remarkable (though transient) clinical response to a single dose of virus^[108]^. Since then, several studies have been published on various NDV-modified cancer vaccines, oncolytic NDV as a monotherapy, and combinatorial approaches including NDV with checkpoint inhibitors, reviewed thoroughly elsewhere by Malogolovkin *et al.*^[66]^. Currently, there is an ongoing phase 1 trial on NDV expressing IL-12 in combination with Durvalumab (an anti-cPD-L1 monoclonal antibody), the preliminary efficacy of which remains to be seen (AstraZeneca 2022 - new ref)^[109]^. Many of these trials reveal significant benefits of NDV in the clinic; however, only a subset of patients responded to treatment. This highlights the importance of finding predictors that will aid in selecting patients most likely to benefit from viral treatment, and in the rational design of recombinant viruses and combinatorial approaches that will overcome resistance.

## INFLUENZA A VIRUS AS AN ONCOLYTIC THERAPY

As the OVs therapy field advances, the available arsenal of oncolytic platforms continues expanding. Among them, Influenza A Virus (IAV) enters the stage as a promising therapeutic candidate. Belonging to the Orthomyxoviridae family, IAV has a broad host range in comparison to subtypes B, C, and D^[110]^. The virus is enveloped and its genome consists of 8 negative-sense single-stranded RNA segments^[111]^. IAV is known to primarily infect respiratory epithelial cells (including Type 2 pneumocytes) followed by immune cells such as macrophages and dendritic cells^[112]^. IAV has been studied extensively in the context of its ability to cause acute disease in humans, ranging from minor to severe illness^[113]^. This may call into question the safety of IAV as an oncolytic, but the large-scale use of live attenuated vaccines in humans attests to the ability to mitigate and answer such a concern^[114]^. This also presents the advantage of already established platforms and systems for manufacturing and mass production of IAV treatments.

Avian strains, such as H5N7, have proven to be safe and effective at targeting cancer types expressing alpha-2,3-lined glycan receptors, like pancreatic ductal adenocarcinoma (PDA)^[115]^; however, extending the use of different IAV strains as cancer therapeutics requires, to a certain extent, the modification of the inner biology of the virus through reverse genetics techniques. Truncation of the *NS* gene, for example, has proven to help with hindering the capacity of the non-structural protein (NS1) to interfere with the immune response of infected cells, making interferon deficient cancer cells prime targets for infection as opposed to normal, healthy cells. For an added measure of safety, (I); swapping the packaging sequences of NS and HA genes and eliminating the intrinsic packaging sequences of these two segments, a procedure known as *rewiring*, has been shown to eliminate the possibility of aberrant reassortment with other IAV in treated patients (II)^[116]^. IAV’s oncolytic candidacy hinges not only on its cytolytic ability but also on its observed ability to elicit an immune response. This response has multiple arms, including induction of immunogenic cell death (ICD) pathways^[60]^. While there are no clinical trials focusing on oncolytic Influenza A Virus yet, preclinical research, typically focusing on the immunogenicity and arming ability of oncolytic IAV, is ongoing. Another facet is the activation of and interaction with innate and adaptive cells in the tumor microenvironment. For example, oncolytic IAV has shown efficacy in provoking T cell response to specific tumor-associated antigens^[62]^. Finally, IAV dNS1viruses, lacking the *orf* (open reading frame) for the NS1 protein, have been highlighted in such research, not only for their potential for targeting specificity but also for potent immunogenicity. This network of response could potentially invigorate the dysregulated and sometimes dampened immune landscape maintained by the hostile tumor microenvironment.

### IAV’s direct effects: induction of immunogenic cell death pathways

IAV infection has been shown to induce a variety of cell death pathways including apoptosis, necroptosis, pyroptosis, and PANoptosis^[117–119]^ [Figure 2]. Depending on the phase of infection, IAV is known to both inhibit and induce apoptosis and the NS1 protein is a key player in this matter. For the case of apoptosis inhibition, one study looked at induction of apoptosis using wild-type influenza A/PR/8/34 and its mutant consisting of a deleted NS1 gene (dNS1) in IFN-competent MDCK cells and IFN-deficient Vero cells^[120]^. Although the dNS1 strain proved to be more lethal in MDCK cells, apoptosis occurred to the same degree with both viruses in the Vero cells, suggesting a relationship between apoptosis and IFN competence. The link between IAV-induced apoptosis and immune stimulation is that some aforementioned pathways also lead to activation of the transcription factor NF-kβ, which regulates cytokine production^[121]^. Furthermore, while activation of apoptosis is the general fate of non-transformed cells infected by IAV, in cancer cell lines, the antiviral response to IAV could lead to different cell death outcomes depending on the genetic background of cancer as well as the viral strain. IAV has been shown to induce apoptosis, as opposed to pyroptosis, in infected A549 lung carcinoma cells^[119]^. A549s treated with caspase 3 inhibitors underwent less cell death after 36 hours of infection; this was not seen with caspase 1 inhibitors. Interestingly, in the same study, a precancerous cell line (PL16T) showed a shift from apoptosis to pyroptosis over time. Another study showed Bax-mediated induction of apoptosis in A549 cells infected with a mutant PR8 strain (PR8-SH3-mf-1) containing 3 point mutations in the NS1 protein^[122]^. In this case, it was determined that the mutant, which was not functionally null but rather failed to activate PI3K/Akt, which was determined to negatively regulate JNKs, kinases known to upregulate both the intrinsic and extrinsic apoptotic pathways^[123]^.

Necroptosis and pyroptosis are more lytic forms of cell death and considered proinflammatory. Although necroptosis has been implicated in tumorigenesis, it has been shown to be capable of inducing anti-cancer immunity through the release of DAMPS, activation/maturation of dendritic cells, cross-priming of cytotoxic T-cells, *etc*.^[124–126]^. A recent study showed RIPK3, regulated by TRIM28, was associated with increased cytokine production and immune cell infiltration^[127]^. Both pathways, during IAV infection, can be mediated by the inflammasome. Nod-like protein 3 (NLRP3), along with ASC and caspase 1, is integral to the inflammasome, specifically in myeloid cells during infection^[128]^. Stimulation based on proton influx from the M2 viral protein leads to conformational changes in the inflammasome and, eventually, production of proinflammatory factors. In the context of the tumor microenvironment, NLRP3 inflammasome activity is associated with multiple ICD paths^[129]^. Recent studies have begun to look at the crosstalk between these pathways during IAV infection [Figure 2]. IAV is speculated to produce Z-RNA (left-handed dsRNA) as a possible PAMP^[130]^. During influenza infection, ZBP1 acts as a proximal sensor necessary for activating the NLRP3-inflammasomes, recruiting RIPK3 and caspase 8, and also leading to caspase 1 mediated production of IL-1β and IL-18 and activation of Gasdermin D which leads to pyroptosis. ZBP1 senses Z-RNA during IAV infection and recruits RIPK3 which activates MLKL in the nucleus, contributing to creating a pore in the nuclear envelope and later in the plasma membrane, thus resulting in necroptosis^[131,132]^. IAV Z-RNA sensing has been speculated to trigger the formation of the PANoptosome, a large complex that allows for crosstalk between and induction of apoptosis, necroptosis, and pyroptosis. This complex is composed of RIPK3, RIPK1, caspase-6, caspase-8, ASC, NLRP3, and caspase-1^[60]^. Crosstalk within this complex can possibly aid in more robust and intricate ICD in cancer cells.

### IAV’s indirect effects

Response to IAV infection must be preceded by viral recognition. Toll-like receptors, RIG-1 receptors, and NLRP3 are three major axes of recognition of IAV viral RNA TLR-3 and TLR-7 are key endosomal RNA sensors that recognize dsRNA and ssRNA, respectively. TLR-7 stimulates Th17 cells differentiation^[133]^. Th17 cells are considered more controversial in terms of their pro- and/or antitumor phenotype^[134]^. Therefore, in the future, it is important to study the effect of stimulation of these sensors on antitumor immunity in the context of oncolytic viruses. TLR-3, in the context of both IAV infection and the tumor microenvironment, has a multifaceted role in disease progression and protection against disease pathogenesis. During IAV infection, TLR-3 stimulation leads to activation of TRIF, which promotes activation of NF-kβ and IRF-3, resulting in production of proinflammatory cytokines and type 1 interferon, respectively^[112]^. Both *in vivo* and *in vitro* studies have shown the presence and importance of stimulation of TLR-3 during flu infection as it relates to immune response and pathogenicity^[135,136]^.

What does viral TLR-3 activation mean for the tumor microenvironment? One example of TLR-3 stimulation, mainly in Batf3-positive dendritic cells and tumor-associated macrophages (TAMs), has been noted for its induction of caspase-3 mediated apoptotic pathways in Non-Small-Cell Lung Cancer (NSCLC)^[137,138]^. Furthermore, a study has shown that stimulation of TLR-3 with poly I:C in MC38 and THP-1 cells leads to upregulation of CD86, CD80, CD40, and iNOS on M2a and M2c macrophages. This alluded to a conversion of M2 to M1 phenotypes and was validated in both mouse and human cell lines. Subcutaneous treatment of tumors with TLR-3 ligand induces tumor regression, the mechanism largely dependent on IFN-α_β_ signaling. In addition to the proinflammatory effects of TLR-3 stimulation, TLR-3 stimulation in a human prostate cancer cell line (DU145) by IAV expressing IL-24, which is typically responsible for activation of STAT1 and STAT3, was shown to induce apoptotic cell death^[139]^. Apoptosis was mediated through the formation of a “TLR-3-associated death-inducing signaling complex, (TLR-3-DISC)”, where pro-caspase 8 is cleaved to induce apoptosis. IL-24 seemed to alter the TLR-3 DISC), as cFLIP, which was noted to be part of the complex when cells were infected with wild-type virus, was absent with the addition of virus expressing IL-24. Specific isoforms of cFLIP block induction of apoptosis. In addition to the absence of cFLIP, cIAP1 (baculoviral IAP repeat-containing protein 2) was downregulated. cIAP1 is a known therapeutic target based on its anti-apoptotic activity in relation to caspase-8 complexes and RIPK1 activity^[140,141]^. Although IL-24 does seem to downregulate viral replication, which could raise concerns for clinical translation, the study showed that the presence of the virus particles alone was sufficient to induce this effect.

As mentioned before, IAV infection in the tumor microenvironment has demonstrated the ability to elicit responses from innate immune cells and to modify their phenotypes. A study on IAV infection of NSCLC demonstrated reprogramming of M2-like macrophages into M1-like macrophages^[61]^. It was hypothesized that since the given NSCLC model has the propensity to develop gain-of-function mutations or upregulation of effectors in the Ras/Raf/MEK/ERK signaling cascade, IAV infection, which seems to be dependent on parts of these pathways, would be able to differentially target these cells, as seen in a previous paper^[142]^. Interestingly, infection in Raf-BxB-expressing tumors of interest yielded lower viral loads after treatment. Alveolar macrophages, after treatment, seem to adopt a phenotype with MHCII+, iNOS+, TNF-α expression. Macrophage phenotype conversion occurred early during infection, indicating that IAV can stimulate a rapid and robust immune response. This may explain why there was a reduction in viral replication and resultant viral loads in Raf-BXB tumor-bearing mice. In support of this idea, depletion of alveolar macrophages led to restoration of viral replication in tumor-bearing mice. Overall, M2 to M1 conversion in the TME was only partial, highlighting the fact that tumors are heterogeneous and can exert immunosuppressive phenotypes that are resistant to oncolytic mediated immunostimulation.

IAV infection has displayed the ability to enhance antitumor immunity by enhancing the activity of tumor antigen-specific lymphocytes. For example, one study shows the upregulation of cross-priming of CD8+ T cells due to IAV infection in SV40-transformed fibroblast cells expressing an oncogenic protein^[62]^. Enhanced T-cell priming and CD8+ T cell expansion proved to be the result of TLR-7 dependent and MyD88 mediated interferon signaling. Given that this mechanism is dependent on TLR-7 and not on TLR-3, it follows that enhanced T-cell responses may be dependent on sensing of IAV’s single-stranded genome. pDCs cells were shown to aid CD8+ dendritic cells in cross-presentation of the tumor-associated antigen when co-cultured, along with CD8− dendritic cells. Enhanced cross-priming, through these mechanisms, is attributed to increased IFN signaling and proinflammatory factors.

### dNS1 IAV oncolytic platform

The NS1 protein is multifunctional but is best known for its contribution to innate immunity evasion during IAV infection. NS1 exists as a homodimer with a RNA binding domain (RBD) and effector domain that interacts with host protein^[143]^. A host cell typically adopts an antiviral state upon infection via mechanisms such as secretion of IFNα/β which leads to expression of ISGs. NS1 is known for not only inhibiting interferon production, but also antagonizing ISGs, regulating apoptosis, and suppressing host gene expression^[143]^. The n-terminus containing the RBD is responsible for competing with sensors such as PKR and RIG-I for dsRNA to prevent downstream IFN production and ISG expression^[144]^. The c-terminus domain binds to proteins such as CPSF (cleavage and polyadenylation specificity factor) and poly(A)-binding protein II (PABII) to intercept cellular pre-mRNA processing^[145]^. These properties make genetic ablation of NS1 an ideal therapeutic approach in the field of vaccine development. One study tested an attenuated NS1-mutant vaccine on pig-tailed macaques and saw increased CD4+ T-cell proliferation in the lung tissue^[146]^. Increased cytokine release during NS1 mutant-IAV infection has also been shown to be NF-kβ dependent based on the increase of IFN-β, CXCL10 and GPR109B (also known as HCA_3,_ Hydroxycarboxylic acid receptor) gene expression^[145]^.

The immunogenic nature of NS1-mutant IAV makes it an attractive option for oncolytic virotherapy, one reason being that dNS1 viruses grow selectively in interferon deficient cancer cells^[147,148]^. Additionally, dNS1 oncolytic strains have been shown to promote the reinvigoration of tumor-infiltrating immune populations. DCs infected with truncated- and deleted-NS1 strains have shown increased activation based on CD86 expression^[149]^. Also, the observed increase in CD86, CD83 and MHC-II molecules indicated an increased potential for T-cell priming. This same study also determined c-terminus deletion to be enough to yield these results. Another study that used a partially deleted (n-terminus) NS1 (NS1–99) virus versus a fully deleted NS1 mutant in SK-MEL1 melanoma tumors in SCID mice observed that the NS1–99 virus produced greater tumor ablation than the other strain^[147]^. This group would later go on to speculate that this result might be due to insufficient replication using the completely deleted NS1 strain. Using a partially deleted NS1 strain expressing IL-15 in IFN-competent B16 mouse melanoma resulted in high levels of type I IFN^[150]^. Other studies have highlighted cases in which fully deleted NS1 viruses seem to lead to more potent expression of proinflammatory factors than partially deleted viruses^[151,152]^. This highlights the importance of considering cancer type and virus strain specificity as it relates to optimizing oncolytic efficacy.

## DISCUSSION

Despite therapeutic innovations becoming accessible to the oncological community in recent years, cancer is still the leading cause of death worldwide. The latest report by the WHO has predicted that, in 2040, 29 million people will be diagnosed with cancer and 16 million people will die from the disease that year alone (Available from: https://gco.iarc.fr/). This forecast highlights not only the change in patient demographics and increased diagnosis but, most importantly, the prevalence of the disease due to the lack of effective treatments. As today, breakthrough technologies such as immunotherapy, Chimeric antigen receptor T (CAR-T) cells and personalized cancer vaccines are still constantly refined to maximize their efficacy and applicability and to reduce the economic burden for the patient. With an estimated 40% of the worldwide population at risk of undergoing cancer, there is an urge for targeted, effective, and affordable therapies.

The significant presence of different OV platforms in clinical trials, especially over the last decade, confirms the recognition of OVs as promising alternative therapy by the broader oncology community^[153]^. Still, the number of early-stage human trials that have not been followed up indicates the need for rationally designed cancer-specific OV-based strategies.

As the research on virotherapy advances, the immunostimulatory features of a given OV platform are displacing in relevance other desired properties that were prioritized before at the time of selecting a particular OV platform, such as the replication capacity of a given virus. In that matter, on December 2022, the FDA approved the first replication-deficient recombinant adenovirus-based cancer therapy, Adstiladrin (nadofaragene firadenovec-vncg: dsDNA), for adults undergoing non-muscle-invasive bladder cancer with carcinoma *in situ*^[154]^. While referred to as novel gene therapy, Adstiladrin is nevertheless a localized virotherapy that takes advantage of the enhanced immunostimulatory capacity elicited by an adenovirus vector carrying interferon alpha-2b as a payload.

The approval of Adstiladrin represents an important milestone in the use of viruses to treat cancer, officially extending their applicability in the clinic as Vectors for gene therapy, and opening the way to other replication-deficient recombinant OVs (rOVs) platforms to be reconsidered for this clinical application. Gene therapy is not the only therapeutic discipline in which OVs’ irruption improves treatment possibilities and outcomes: OVs have been shown to be a good match for CAR-T cells-based therapies, improving its responses in solid tumors through TME remodeling and/or maximizing T cell functions^[155–158]^. Local delivery of some therapeutic OVs, such as NDV, has demonstrated their suitability for *In situ* cancer vaccination, leading to complete durable responses without the need to identify neoantigens beforehand^[39,159–161]^. OVs serving as neoadjuvants have been shown to sensitize and improve responses to radiotherapy and chemotherapy^[162–165]^. As of today, manipulation of immune responses by OVs at the TME is at the base of the latest approaches being tested in the clinic, thus involving rOVs expressing cytokines and chemokines in combination with immune checkpoint blockade (ICB) agents, aiming to counteract the immunosuppressive nature of the TME, improving T cell functions while maximizing the killing and immunostimulatory capacities of the virus (Available from: ClinicalTrials.gov).

Here, we presented a comprehensive review of the immunological responses to ssRNA (−) OV platforms, with a closer look into NDV and IAV. For both types of OVs, there have been extensive efforts to understand the immune biology behind their therapeutic potential, allowing for the design of rOVs and combination therapies that could further enhance their contribution to the antitumor response. rNDVs have been developed to enhance their therapeutic properties such as induction of ICD, or to modify interactions in the TME through the expression of cytokines, chemokines and immune checkpoint inhibitors (ICIs). rIAVs have been developed to express ICIs such as the anti-CTLA-4 antibody or cytokines like IL-15, with the aim of facilitating the activity of cytotoxic cells at the TME. More recently, Masemann *et al.* observed synergistic effects of IAV infection combined with B7-H3 and PD-L1/2 inhibitor treatments when applied to NSCLC [Table 2]^[61]^.

In the nearest future, breakthrough oncolytic virotherapies will be designed taking into consideration the immunological responses that a given viral platform can induce in a given type of tumor, attending to the genetics and immune context of the TME and the characteristics of the patient - ssRNA(−) OVs aim to be at the forefront of the next generation of cancer virotherapeutics.

## Figures and Tables

**Table 1.** Summary of the innate and adaptive immune responses to oncolytic ssRNA(−) viruses

Virus	Innate immune responses	Adaptive immune responses

Newcastle disease virus	Inflammasome activation via NLRP3^[15]^ Type I IFN activation^[16,17]^ Release of DAMPs^[18,19]^ NK activation via HN binding^[20,21]^ DC maturation^[18,22]^ M1 macrophage polarization^[22]^	Cross-presentation by DC1^[22]^ Increased CD8+/CD4+ T cell infiltration & activation^[23,24]^ Decrease in T-regs^[23]^
Sendai (HVJ-E)	SAMD9 expression via type-I IFN^[25]^ Stimulates RIG-I/MAVS in cytoplasm, enhancing Noxa and TRAIL via IRF-3 and IRF-7^[26]^ Promotes DC maturation, inducing production of IFN-α (especially IFN-α1a), -β, and -y, TNF-α and IL-6; causes DC tumor infiltration^[27]^ Recognition of viral RNA genome by TLR-7 and -8 in the endosome Activation of NK cells by CXCL-10^[28]^ Removal of sialic acid receptors from cells^[29]^	IL-6 enhances the proliferation and activation of cytotoxic T-lymphocytes (CTLs) by suppressing the activity of regulatory T-cells (T-regs)^[30]^ Improves presentation of antigens MHC-I and MHC-II^[29]^
Measles	Increased TRAIL-mediated cytotoxicity by myeloid and plasmacytoid DCs^[31]^ Modulation of macrophages towards an antitumor phenotype through macrophage repolarization^[32]^ Activation of neutrophils (secretion of IL-8, TNF-α, MCP-1, and IFN-α, TRAIL expression, and degranulation)^[33]^ Sensing of viral RNA by PRRs, RIG-I and MDA-5 triggering production of type 1 interferon (IFNα/β)^[34]^ Induction of ISGs involved in anti-viral defense and apoptosis signaling by IFN binding to JAK/STAT pathway^[35,36]^	Immunogenic cell death^[37]^ Induction of distinctive immunopeptidome^[38]^ Promotion of cross-priming of antitumor T celkesponses by conventional and plasmacytoid dendritic cells^[39,40]^ Enhanced production of IFNa and cross-presenting of tumor antigens to CD8+ T-cells by Plasmacytoid DCs (pDCs)^[41]^ Activation of DCs by upregulating costimulatory surface activation markers CD80 and CD86 ^[42]^
APMV-4	Strong Type I IFN response^[43]^	Shown to confer immunological memory after complete remission of tumors *in vivo*^[43]^
Vesicular stomatitis virus	Infiltration of neutrophils and NK cells^[44,45]^ and induction of type III IFN Mediation of pDC maturation and activation through TLR-7, leading to induction of IFNa and priming of CD8+ T cells^[44–46]^ Induction of type I IFN expression through TLR-4, TLR-3, TLR-7, and TLR-13 and RIG-I^[44–48]^ Upregulation of MHC class II, CD80, CD86, CD40, which all lead to improved antigen presentation, as well as induction of high levels of type I IFNs^[49–52]^ Release of TAAs, PAMPs and DAMPs, induction of ICD markers, such as ecto-CRT, HMGB1, ATP, and Hsp70 and Hsp90^[53]^ Release of IL-28 in TME, tumor cells display NK cell ligands; NK, activation and cytotoxicity^[54]^	Induction of tumor-specific CD8+ T cells that are induced following the release of tumor-associated antigens^[55,56]^
Maraba virus MG1	Increased induction of DCs and NK effector cells secreting IFN-γ or granzyme B^[57]^ Increased expression of chemokines like CCL5, CXCL11^[58]^ Production of IFN-γ reliant on the receptor IFN-αR1^[58]^	Greater infiltration of MG1-infected tumors by T-cells *in vivo,* a migration dependent on CXCR3, the target receptor of the Th1/Tc1- associated chemokines CXCL9, 10, and 11^[59]^
Influenza A virus	Induction of PANoptosis^[60]^ M2 to M1 polarization^[61]^ pDC maturation^[62]^ DCs cross-presentation of TAA to CD8+^[62]^	Cross priming of CD8+ T cells^[62]^ Increased expansion of CD8+ T cells^[62]^

NLRP3: NOD-, LRR- and pyrin domain-containing protein 3; IFN: interferon; DAMP: damage-associated molecular pattern; NK: natural killer; HN: hemagglutinin-neuraminidase; DC: dendritic cell; M: macrophage; CD: cluster of differentiation; T-regs: regulatory T cells; SAMD9: sterile alpha motif domain-containing protein 9; RIG-I: retinoic acid-inducible gene I; MAVS: mitochondrial antiviral-signaling protein; TRAIL: TNF (tumor necrosis factor)-related apoptosis-inducing ligand; IRF: interferon regulatory factor; TNF-α: tumor necrosis factor alpha; IL-6: interleukin 6; RNA: ribonucleic acid; TLR: toll-like receptor; CXCL: CXC chemokine ligand; CTL: cytotoxic T-lymphocytes; MHC: major histocompatibility complex; MCP-1: monocyte chemoattractant protein-1; CXC: chemokine ligand; PRR: pattern recognition receptor; MDA5: melanoma differentiation-associated protein 5; ISG: interferon-stimulated gene; JAK/STAT: Janus kinase (JAK) - signal transducer and activation of transcription (STAT); pDC: plasmacytoid dendritic cell; TAA: tumor-associated antigen; PAMP: pathogen-associated molecular pattern; ICD: immunogenic cell death; CRT: calreticulin; HMGB1: high mobility group box protein 1; AT: adenosine triphosphate; HSP: heat shock protein; TME: tumor microenvironment; CCL: CC chemokine ligand; IFN-αR1: interferon-alpha receptor 1; Th1: Type 1 helper; Tc1: Type 1 (CD8+) T cells.

**Table 2.** Recombinant NDV and IAV viruses for oncolytic therapy

Virus	Payload	Therapeutic enhancement	Refs.

NDV	IL-2	TME remodeling; ↑TILs	[166]
	IL-15	TME remodeling; ↑+TILs; ↑+NK	[167]
	IL-12	TME remodeling; ↑+TILs	[168]
	FAS	ICD, +TILs	[81]
	Anti-CD28-IL-12	TME remodeling; ↑+TILs	[169]
	IL-2-TRAIL	TME remodeling; ↑+TILs	[170]
	OX40L	TME remodeling; +Th2 responses	[171]
	ICOS	↑+TILs	[172]
	GM-CSF	↑+DCs	[173]
	MIP3α	TME remodeling; ↑+ APCs; ↑+TILs	[174]
	IAV-CTLA-4	TME remodeling; +TILs;	[175]
IAV	NS1-GM-CSF	↑+APCs	[176]
	NS1-IL-15	↑+TILs; ↑+NK	[149,151]
	NS_116_-GFP/AE	Tumor target: elastase-dependent	[177]

↑Increased recruitment; +Enhances cell function. AE: Elastase; APCs: antigen-presenting cells; CTLA-4: cytotoxic T cell related protein-4; ΔNS1: delta-NS1; FAS: human Fas receptor; GFP: green fluorescence protein; GM-CSF: granulocyte-macrophage colony-stimulating factor; ICD: immunogenic cell death; ICI: immune checkpoint inhibitors; ICOS: inducible T cell costimulatory (CD278); IL: interleukin; MIP3α: macrophage inflammatory protein 3; NK: Natural killer cell; PD-L1: programmed cell death ligand 1; TILs: tumor infiltrating lymphocytes; Treg: regulatory T-cells; TRAIL: TNF-related apoptosis-inducing ligand.
